# Smoking and epidemics of respiratory infections

**DOI:** 10.2471/BLT.20.273052

**Published:** 2020-10-28

**Authors:** Freddy Sitas, Ben Harris-Roxas, Debbie Bradshaw, Alan D Lopez

**Affiliations:** a Centre for Primary Health Care and Equity, School of Population Health, Level 3 AGSM Building, University of New South Wales-Sydney, High Street, Kensington, Sydney, NSW 2052, Australia.; b Burden of Disease Research Unit, South African Medical Research Council, Tygerberg, South Africa.; c Melbourne School of Population and Global Health, The University of Melbourne, Victoria, Australia.

The causative role of smoking in respiratory infectious disease has been well documented in reports of the United States Surgeon General,^1^ but remains underappreciated because the main focus of the health community is on the long-term effects of smoking on chronic diseases. Global estimates from 2017 suggest that among adults aged 35–74 years, there were about 490 million incident cases and 1.34 million deaths due to infectious respiratory diseases.^2^ Of these, 22.5% (approximately 300 000 deaths) were attributed to smoking. 

This century we have seen pandemics of Middle East respiratory syndrome (MERS), severe acute respiratory syndrome (SARS), H1N1 influenza virus and now coronavirus disease 2019 (COVID-19). By October 2020, COVID-19 had caused over a million deaths, already accounting for 2% of all-cause global mortality. These respiratory pandemics will add to the 1 billion deaths expected to occur from smoking this century if current smoking trends persist.^3^

Smoking increases the risk of death in the context of known respiratory infectious diseases by increasing the number of incident cases and by worsening case fatality rates, as documented below. This increased risk needs to be considered during the current COVID-19 pandemic.

Evidence for smoking increasing incidence of infectious respiratory disease has been reviewed extensively.^1^ A recent review of 27 studies^4^ shows that current smokers double their odds of community-acquired pneumonia (odds ratio, OR: 2.2; 95% confidence interval, CI: 1.7–2.8). In a review of 20 studies of relative risk of tuberculosis in relation to current smoking,^5^ 14 showed a positive association. One study showed a twofold likelihood (OR: 1.9; 95% CI: 1.1–3.3) of daily smokers (versus occasional, former or non-smokers) reporting flu-like symptoms at the time of the H1N1 influenza epidemic,^6^ while another found an OR of 3.1 (95% CI: 1.1–9.2) of MERS cases reporting smoking.^7^ Population-based studies on COVID-19 incidence among smokers are emerging. In a household survey in the United Kingdom of Great Britain and Northern Ireland, current smokers older than 17 years were more likely to report COVID-19 symptoms compared to those who had never smoked (OR: 1.3; 95% CI: 1.0–1.7), with non-significant results from e-cigarette use.^8^ Among young people in the United States of America aged 13–24 years, compared to never users, ever-smokers of both e-cigarettes and cigarettes (dual-users) were seven times (OR: 6.97; 95% CI: 2.0–24.6), ever users of e-cigarettes only were five times (OR: 5.1; 95% CI: 1.8–14.0), and past 30-day dual-users were 6.8 times (95% CI: 2.4–19.6) more likely to self-report a diagnosis with COVID-19.^9^ Such recent emerging results and evidence from other infectious respiratory conditions suggest the outcome of the association between COVID-19 and smoking will be broadly similar to what has been previously observed in other infectious respiratory diseases.

The evidence regarding smoking and mortality from infectious respiratory diseases is also well established.^1^ However, the relative importance of infectious respiratory disease and smoking was made more apparent when large studies in low- and middle-income countries such as India and South Africa^10^ showed tuberculosis and infectious respiratory diseases to be the leading causes of death from smoking.

While the science regarding the role of smoking and COVID-19 has been evolving, sufficient evidence to measure the impact reliably is not yet available. A meta-analysis of 16 studies shows that smokers are 50% more likely to have more severe COVID-19 symptoms (combined OR: 1.5; 95% CI: 1.2–1.9), and a meta-analysis of five studies on COVID-19 and risk of mortality shows a positive, non-significant association (OR: 1.5; 95% CI: 0.8–2.7).^11^

Given this growing body of evidence, what can health authorities do to better understand the burden of smoking and minimize its risk in the COVID-19 era and in future pandemic situations? Clearly, health authorities need to develop better messaging and approaches to identifying, funding and supporting priority research to fill key knowledge gaps as well as investing in cost-effective research infrastructure that will reliably monitor excess risks of COVID-19 incidence and deaths in current, former and non-smokers. In addition, we need to improve two action areas. 

First, messaging: rather than focusing on debates about causality of a new agent, governments and health authorities can make definitive statements now on the impact of smoking on infectious respiratory disease in general. Seasonal infectious respiratory diseases and other respiratory pandemics may present opportunities for smoking cessation. General practitioners or health staff can use the opportunity of a seasonal or pandemic respiratory disease to identify and advise patients who smoke to quit. There may also be opportunities to embed smoking cessation information and interventions into testing procedures and hospital discharge planning processes, which would have a range of health co-benefits.

Second, data infrastructure: recording of smoking status at hospital admission is already poor.^12^ Collecting accurate epidemiological information on smoking (or any lifestyle and/or environmental information) from acutely ill patients, especially in pandemic situations, is therefore not ideal. While smoking status collection in health records needs to be enhanced, smoking status in this context needs cautious interpretation. Smokers may cease smoking during respiratory incidents and inadvertently report themselves as non-smokers. Because of real or perceived stigma, smokers attending a health service with acute respiratory symptoms may well report themselves as non-smokers if they feel they may be triaged out of treatment. Large cohort studies could yield sufficient COVID-19 cases (notifications, hospitalizations, deaths) to achieve statistical power, so these should be used to provide information about risk and environmental factors increasing incidence of COVID-19 disease and death. Countries with good mortality statistics can monitor their causes of death to understand the complex ways in which COVID-19 can cause excess deaths. Countries such as China (Tianjin) and South Africa that adopted questions on smoking on their death notification forms^10^ can benefit from a cost-effective and enduring source of information on risks of smoking for specific causes of death, for this and future pandemics. Retrospective surveys of next-of-kin of recently deceased individuals can enquire about tobacco use status of their deceased relative – a limited number of modest lifestyle questions can be asked with robust results.
